# Implications of COVID-19 Pandemic on Evolution of Diabetes in Malaria-Endemic African Region

**DOI:** 10.1155/2020/8205261

**Published:** 2020-10-08

**Authors:** Samuel Acquah

**Affiliations:** Department of Medical Biochemistry, School of Medical Sciences, College of Health and Allied Sciences, University of Cape Coast, Cape Coast, Ghana

## Abstract

The coronavirus disease 2019 (COVID-19) pandemic continues to cause havoc to many countries of the globe, with no end in sight, due to nonavailability of a given vaccine or treatment regimen. The pandemic has so far had a relatively limited impact on the African continent, which contributes more than 93% of global malaria burden. However, the limited burden of COVID-19 pandemic on the African region could have long-term implications on the health and wellbeing of affected inhabitants due to its malaria-endemic status. Malaria causes recurrent insulin resistance with episodes of infection at relatively low parasitaemia. Angiotensin-converting enzyme 2 (ACE2) which is widely distributed in the human body is implicated in the pathogenesis of malaria, type 2 diabetes mellitus (T2DM), and COVID-19. Use of ACE2 by the COVID-19 virus induces inflammation and oxidative stress, which can lead to insulin resistance. Although COVID-19 patients in malaria-endemic African region may not exhibit severe signs and symptoms of the disease, their risk of exhibiting heightened insulin resistance and possible future development of T2DM is high due to their prior exposure to malaria. African governments must double efforts at containing the continued spread of the virus without neglecting existing malarial control measures if the region is to avert the plausible long-term impact of the pandemic in terms of future development of T2DM.

## 1. Introduction

**Figure 1:**
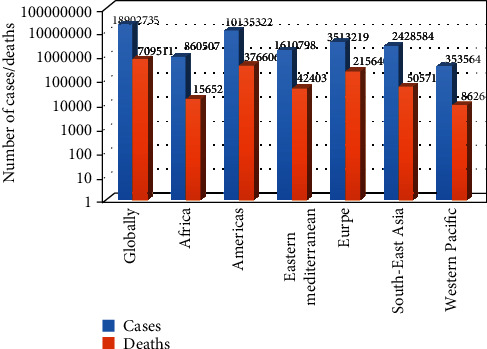
WHO regional number of COVID-19 cases/deaths as of 7^th^ August, 2020 at 10 : 00 CEST (logarithmic scale).

The novel coronavirus disease (COVID-19) pandemic that started in Wuhan, China, in December 2019, has now affected 216 countries and territories as of 7^th^ August, 2020. COVID-19 had infected 18,902,735 people worldwide with 709,511 deaths (Figure 1) with no sign of a definite treatment as of 10 : 00 CEST on 7^th^ August, 2020 [1]. The global epicenter of COVID-19 appears to move from one country to the next with Brazil currently holding the baton as the global epicenter. The pandemic continues to exhibit inter- and intracontinental variations per million indices in terms of number of cases, deaths, and tests with no country emerging as the leader in all the three indices. This trend clearly shows the varied resilience of country-specific response to the pandemic. In terms of absolute numbers, the WHO American region appears to be the most affected followed by Europe with Africa being the least affected region of the globe after the Western Pacific region. However, when the available data are presented per million population, the European region of the globe becomes the most affected. Although the pandemic is still raging, the interim differences in deaths and tests among countries and continents can be ascribed to variations in population dynamics, effectiveness of health systems and policies, and economic factors. Since the pandemic appears to influence every aspect of human endeavor, it is pertinent that its impact is assessed from multidimensional perspectives. The current review aims at discussing the possible impact of COVID-19 pandemic on the evolution of diabetes in malaria endemic regions of the globe.

## 2. COVID-19, Malaria, and Diabetes

Whereas the COVID-19 pandemic and diabetes are truly global in distribution, malaria appears to be an African problem (Table 1). The African continent remains the hub of global infectious diseases, as there is no historical account of any infectious agent of humanity [2–4] that fails to mention the African continent. It is, perhaps based on some of this historical premise and its unique settlement, that the COVID-19 pandemic is projected by the World Health Organisation (WHO) to infect 29 million to 44 million Africans with associated 83,000 to 190,000 deaths if containment measures put in place by various African governments fail [5]. Additionally, it is predicted that the duration of COVID-19 infection in the African region could be longer than expected if African governments do not take appropriate proactive measures [5]. Indeed, the COVID-19 pandemic continues to cause havoc to many countries globally with no end in sight. As such, the exact impact of COVID-19 on any given country is not yet fully known. Fortunately, various African governments have put in place pragmatic measures to contain the spread of the virus to avert the gloomy predictions. If the measures in place work effectively, Africa will enviably remain the second least affected region of the globe in terms of COVID-19 morbidity and mortality. However, the potential long-term impact of the COVID-19 pandemic on the health burden of Africans in general and the sub-Saharan African region in particular cannot be overemphasized. This has become critical due to the generally mild nature of COVID-19 cases on the African continent. With the continent already responsible for over 93% of global malaria burden and associated deaths in 2018 [6], the presence of the COVID-19 pandemic does not only increase the infectious disease burden of the African region but could serve as another risk factor to the development of type 2 diabetes mellitus (T2DM) in the region. Indeed, current estimates by the International Diabetes Federation (IDF) predict the African region to experience 173% increase in incidence of diabetes by 2045 compared with a global average of 51% [7]. Also, the proportion of undiagnosed diabetes cases in the African region was 59.7%, being the highest on the globe per the 2019 IDF report [7]. Thus, the proportion of undiagnosed cases of diabetes will increase further in the face of the COVID-19 pandemic globally and most importantly in resource-constraint African region of the globe. Considering the relatively high rate of infectivity and mortality of the COVID-19 pandemic virus, it is only logical that the highest of priority is given in terms of resource allocation to halt the continuous spread of the virus and move towards its elimination from the global health calendar. As such, as resource-constraint African countries direct available resources to combat the pandemic, not much will be left to cater for other competing health needs such as the prevention of malaria and diabetes complications through testing for early diagnosis. Above all, some of the identified pathogenic mechanisms of the virus responsible for the COVID-19 pandemic appear to be similar to some known risk factors to the development of diabetes such as obesity and malaria [7–11].

## 3. Effects of COVID-19 on the Host

The COVID-19 outbreak, which started in Wuhan city in Hubei Province of China, was officially made known to the WHO on December 31, 2019 [12, 13]. Since then, the viral pathogen has spread to 216 countries and territories of the globe. Just like other known coronaviruses [14, 15], the acute respiratory syndrome coronavirus-2 (SARS-CoV-2), responsible for the COVID-19 pandemic, requires a specific receptor, angiotensin-converting enzyme 2 (ACE2), for entry into host cells [16–19]. Indeed, the use of ACE2 as a receptor by coronaviruses has long been reported [20, 21]. ACE2 is a zinc-containing monocarboxypeptidase that catalyzes the conversion of angiotensin I and angiotensin II to angiotensin 1–9 and angiotensin 1-7, respectively [22]. Through angiotensin 1-7, ACE2 ameliorates lung fibrosis, vascular damage, pulmonary hypertension, and pulmonary injury [23–25]. A role for ACE2 has also been recognized in the development of T2DM [26–30] and malaria [31–33]. Angiotensin II is a known blood pressure-inducing peptide that narrows the lumen of blood vessels through persistent contraction, renal retention of water and salt with associated increased resistance, and heightened blood pressure [34]. Angiotensin II is an octapeptide produced by the hydrolytic action of angiotensin-converting enzyme (ACE) on angiotensin I, a decapeptide, which is produced from angiotensinogen by renin. The overall effect of angiotensins I and II is to increase blood pressure. As such, reduced levels of angiotensins I and II or inhibition of their production or activities result in restoration of normotension but may increase susceptibility to malaria [31–33, 35]. Thus, the angiotensin system is critical in health and disease. The main component of the renin-angiotensin system with the capability to link COVID-19, malaria, and diabetes mellitus is the ACE2. The action of ACE2 in reducing angiotensin II levels does not only improve cellular haemodynamics but attenuates antimalarial properties to potentially facilitate infection and inflammation which may increase the risk for development of diabetes [9, 31–33, 35].

In a study involving 518 severe acute respiratory syndrome (SARS) patients and 19 patients with non-SARS pneumonia, Yang et al. [36] reported that binding of SARS coronavirus to its receptor, ACE2, in the pancreas, caused transient type 1 diabetes mellitus through damaged pancreatic islet. This observation made in this previous SARS coronavirus infection suggests that the damage caused by that coronavirus to pancreatic islet was not permanent but reversible. As such, patients who recovered from the disease with time were able to restore normal pancreatic islet function in synthesizing and secreting insulin for glucose homeostasis. Although this may be plausible for the COVID-19 virus, available evidence on the devastative nature of the COVID-19 virus, rather, supports a potential long-term impact on the pathogenesis of diabetes mellitus. The COVID-19 virus is having a huge impact on global health due to its stronger bonding affinity for ACE2 and unique furin cleavage sites [37] and ability to take advantage of several host proteases to facilitate infection [17–19]. ACE2 has been found in various cells and tissues including endothelium, heart, intestinal epithelium, lungs, pancreas, renal tubular epithelium, upper respiratory tract, and the central nervous system [38, 39]. Indeed, symptomatic patients of the COVID-19 virus may present dry cough, diarrhoea, dyspnea, fatigue, fever, headache, myalgia, nasal congestion, nausea, runny nose, sore throat, tastelessness, and vomiting [40–42], reflecting the wide distribution of ACE2 in the human system.

## 4. Effects of COVID-19 and Malaria on Pathogenesis of Diabetes

Hypertension is a known risk factor for T2DM, and both diabetes and hypertension are leading underlying comorbidities for unfavorable COVID-19 prognosis [40, 41]. As earlier indicated, the COVID-19 virus enters cells of susceptible hosts through ACE2 [16–19]. Binding of the virus to ACE2 reduces ACE2 levels and its degradative effects on angiotensin II [43], resulting in accumulating levels of angiotensin II and resultant negative effects on pulmonary and vascular homeostasis [23–25].

Several experimental studies in animals have shown that increased levels of angiotensin II result in abnormal hepatic lipid and carbohydrate metabolism [44, 45]. Other studies have demonstrated the critical role of the renin-angiotensin system in hepatic and adipose tissue inflammation, insulin resistance, and glucose intolerance [46, 47]. Indeed, Santos et al. [48] demonstrated that oral administration of angiotensin-(1-7), a degradative product of angiotensin II, prevented high-fat diet-induced obesity, inflammation, insulin resistance, and glucose intolerance through downregulation of resistin, nuclear factor kappa B (NF-*κ*B), toll-like receptor 4 (TL4), interleukin-6 (IL-6), tumor necrosis factor-*α* (TNF*α*), and mitogen-activated protein kinase (MAPK) levels.

Inflammation and oxidative stress interact in a synergistic manner to promote the development of various health conditions including diabetes [49–51]. To this end, the COVID-19 virus, which reduces ACE2 levels or activities, can potentiate infected individuals for future development of T2DM through low-grade inflammation and insulin resistance. This is very critical in view of several observations that a number of infected individuals do not exhibit any signs and symptoms [52, 53]. Indeed, most of the COVID-19 cases in the African region fall within the mild and asymptomatic category explaining the low hospitalization rate for positive cases in the region. The presence of asymptomatic cases suggests that the virus can induce low-grade inflammatory process which can lead to the development of insulin resistance as the body takes appropriate steps to clear the virus. It has been reported that viral particles are detectable long after recovery from the infection [54], suggesting that the body's inflammatory process can still be engaged as a homeostatic mechanism to ensure normal cellular function. Thus, in both symptomatic and asymptomatic cases, inflammation is triggered and sustained, during and after infection to reestablish homeostasis. Specific inflammatory molecules such as TNF*α*, IL-6, and other inflammatory signaling pathways identified with COVID-19 have been associated with the pathogenesis of type 2 diabetes mellitus [55] and malaria [56] through insulin resistance [57]. As a result, the COVID-19 virus, through its interaction with ACE2 may predispose affected individuals to future development of diabetes associated with low-grade inflammation and insulin resistance.

**Figure 2:**
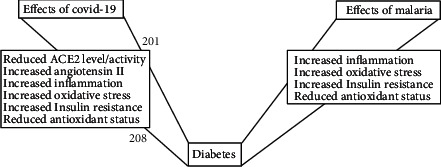
Effects of COVID-19 and malaria on the development of diabetes.

Data from human [9, 58] and animal [8] studies on malaria-induced insulin resistance suggest that insulin resistance can develop within a relatively short period of infection. In addition, once established, treatment of the infection does not reduce the level of the established insulin resistance to the preinfection level. As such, upon subsequent infection even at relatively low parasitaemia, insulin resistance is reestablished, suggesting that the previous infection primed affected cells to develop insulin resistance upon least reexposure. It is therefore plausible that insulin resistance caused by the COVID-19 virus will also create a kind of immunological memory on affected cells, priming them for future development of insulin resistance upon exposure to other infectious agents or other favorable factors. Unlike a typical immunologic memory to a given pathogen whereby a heighted immune response requires reexposure to the specific pathogen, insulin resistance priming does not require reexposure to the same pathogen that primed the cells. As such, virus-induced insulin resistance can prime affected cells for the development of insulin resistance upon later exposure to any other pathogen. This is due to the fact that the cellular factors involved in the insulin resistance development process(es) do not differ necessarily with the kind of pathogen. For instance, IL-6 and TNF*α* released by COVID-19 are also triggered by malaria and obesity as far as inflammation-induced insulin resistance is concerned. Thus, the pathogenic agent could be different but the cellular mechanisms triggered, and the specific players involved in inducing the requisite pathogenic inflammatory processes may be the same. It is based on this fundamental concept that it is speculated that COVID-19-infected individuals who recover from the disease may be prone to future development of insulin resistance and possibly diabetes mellitus upon exposure to favorable factors such as obesity and malaria. This is very crucial for sub-Saharan Africa that is already burdened with various infectious agents and struggling to deal with its increasing trend of diabesity. Considering that the region has the highest proportion of undiagnosed diabetes [7], the relatively low burden of COVID-19 infections on the African continent and the mild and asymptomatic nature of the infections could be seen as a postponed burden of T2DM. As such, the continent should rather intensify efforts at preventing further spread of the COVID-19 virus in order to avert a potential catastrophe in the form of future development of T2DM (Figure 2).

In a recent editorial, Napoli and Nioi [59] postulated that malaria could be playing a protective role in COVID-19 severity on the African continent apart from her relatively young population. This hypothesis was based on the relatively limited number of cases of COVID-19 on the continent and other malaria-endemic regions of the globe together with antiviral properties of some antimalarial drugs. However, the postulated immune protection provided by prior malaria infection does not protect against the negative effects of inflammation. Additionally, various antimalarial drugs employ oxidative stress as a mechanism for parasite clearance [60]. More so, mild inflammation coupled with drug-induced oxidative stress can cause unintended organ damage [61]. Above all, there is no evidence of active malaria being protective against COVID-19. In fact, active malaria, due to compromised immunity of the affected, is rather expected to facilitate COVID-19 infection. On this note, active malaria in COVID-19 patients should rather be treated aggressively to avert worsened outcome of COVID-19. Thus, the mild cases of COVID-19 provide the right environment for low-grade inflammation, coupled with oxidative stress necessary for the development of insulin resistance (Figure 2) and increase the risk for development of T2DM.

## 5. Conclusion

Although Africa remains the second least affected COVID-19 region of the globe, the pandemic still poses a serious health challenge in terms of potential future development of T2DM. This is premised upon mild inflammation associated with mild cases of COVID-19 infection, which can cause insulin resistance and increase the risk for development of T2DM. African governments should continue to intensify efforts at preventing the spread of COVID-19 without losing focus on the need to continue to take steps to control malaria in the region. Such steps have potential to protect the region from undue future development of T2DM.

## Figures and Tables

**Table 1:** Total cases and deaths from COVID-19, diabetes, and malaria in Africa and the world.

Cases/deaths	Diabetes		Malaria		COVID-19	
	Africa	World	Africa	World	Africa	World
Cases	19,000,000	463,000,000	213,000,000	228,000,000	216,999	18,902,735
Deaths	366,200	4,200,000	380,000	405,000	4,874	709,511

Sources: COVID-19 estimates were obtained from WHO Situational Report 200 based on data from national authorities as of 10 : 00 CEST on 7^th^ August, 2020. Diabetes estimates were obtained from the 9^th^ edition of IDF diabetes atlas, 2019. Malaria estimates were obtained from the world malaria report, 2019.
